# Leaf Bleaching in Rice: A New Disease in Vietnam Caused by *Methylobacterium indicum*, Its Genomic Characterization and the Development of a Suitable Detection Technique

**DOI:** 10.1264/jsme2.ME21035

**Published:** 2021-11-03

**Authors:** Khoa Lai, Ngoc Thai Nguyen, Michiko Yasuda, Khondoker M.G. Dastogeer, Atsushi Toyoda, Koichi Higashi, Ken Kurokawa, Nga Thi Thu Nguyen, Ken Komatsu, Shin Okazaki

**Affiliations:** 1 United Graduate School of Agricultural Science, Tokyo University of Agriculture and Technology, Saiwaicho 3–5–8, Fuchu, Tokyo 183–8509, Japan; 2 National Agro-Forestry-Fisheries Quality Assurance Department branch 4, 271 - To Ngoc Van St, Linh Dong ward, Thu Duc district, Ho Chi Minh City, Vietnam; 3 Graduate School of Agriculture, Tokyo University of Agriculture and Technology, Saiwaicho 3–5–8, Fuchu, Tokyo 183–8509, Japan; 4 Department of Plant Pathology, Bangladesh Agricultural University, Mymensingh-2202, Bangladesh; 5 Department of Genomics and Evolutionary Biology, National Institute of Genetics, Shizuoka, Japan; 6 Genome Evolution Laboratory, National Institute of Genetics, Yata 1111, Mishima, Shizuoka 411–8540, Japan; 7 Department of Plant Protection, College of Agriculture and Applied Biology, CanTho University, 3/2 St, Ninh Kieu district, CanTho, Vietnam

**Keywords:** *Methylobacterium*, rice plants, disease, bleaching symptom, LAMP

## Abstract

A new disease in rice that is characterized by leaf bleaching was recently identified in some fields in the Mekong Delta region of Vietnam. The present study was the first to isolate and identify the pathogen of this disease. We confirmed that leaf bleaching symptoms were due to infection with *Methylobacterium indicum* bacteria using molecular biology approaches. A full-length genome analysis of pathogenic *Methylobacterium* strain VL1 revealed that it comprises a single chromosome and six plasmids, with a total size of 7.05‍ ‍Mbp and GC content of 70.5%. The genomic features of VL1 were similar to those of the non-pathogenic *M. indicum* strain SE2.11^T^; however, VL1 possessed additional unique genes, including those related to homoserine lactone biosynthesis. We established a loop-mediated isothermal amplification (LAMP) assay using the unique sequences of VL1 as target sequences for the rapid and simple detection of pathogenic *M. indicum* strains. Our initial evaluation demonstrated that the LAMP assay successfully distinguished between pathogenic and non-pathogenic strains infecting rice plants in a rapid and sensitive manner. The present results provide insights into the pathogenesis and development of control measures for novel rice diseases.

Rice (*Oryza sativa* L.) is one of the most important food crops in the world, particularly in Asia. In Vietnam, rice production plays a pivotal role in the country’s economy, with approximately 80% of farmers growing rice (Nguyen and Singh, 2006). The Mekong Delta in Vietnam includes four million hectares of rice fields, which produce approximately 16.2 million tons of rice, accounting for approximately 90% of the country’s total rice export (Nguyen and Singh, 2006). Therefore, a negative impact on rice production (*e.g.*, disease) may significantly impact the economy and food security of Vietnam and related countries.

In 2010, local farmers in the Mekong Delta reported a new rice disease referred to as leaf bleaching. Symptoms appeared on rice seedlings at the age of 15 to 20 days, and included white coloration on leaves and markedly delayed plant growth, leading to death in the worst cases (Khuyen Nong, 2010). Disease symptoms appeared to differ from those of known rice diseases (Thuan *et al.*, 2006; da Cruz *et al.*, 2013; Ta *et al.*, 2013; Sharma *et al.*, 2017). Limited information is currently available on the causal agents, pathogenic biology, epidemiology, and control measures of this disease.

We recently investigated the diversity of *Methylobacterium* in the phyllosphere of rice cultivated in the same areas of Vietnam in which the new disease was reported (Lai *et al.*, 2020). Specific strains were found to produce unusual symptoms in rice seedlings upon inoculation, including the gradual yellowing of leaves that ultimately developed into bleaching and the appearance of white areas. Plant growth was retarded and affected seedlings succumbed to death in an untimely manner. The symptoms described by Vietnamese rice farmers and those that we encountered appeared to be similar. Therefore, we hypothesized that *Methylobacterium* bacteria may be the causal pathogens of this disease in Vietnam. Although previous studies reported the plant growth-promoting activities of *Methylobacterium* species (Lee *et al.*, 2006; Knief *et al.*, 2012), they have not previously been shown to exert pathogenic effects on rice or other plants.

According to 16S rRNA gene phylogeny, the pathogenic *Methylobacterium* strains that we isolated in our previous study were closely related to *M. indicum* (Lai *et al.*, 2020). *M. indicum* has also been isolated from surface-sterilized rice seeds in India (Chaudhry *et al.*, 2016). The draft genome of non-pathogenic *M. indicum* SE2.11^T^ showed a similarity of only 35.55% with the most closely related type strain, *M. plantani* JCM 14648^T^, by digital DNA-DNA hybridization (dDDH) (Chaudhry *et al.*, 2016). Therefore, the genomic characterization of pathogenic *M. indicum* has yet to be completed. We aimed to analyze the genome of pathogenic *M. indicum* and compare it with those of non-pathogenic strains in order to identify putative pathogenic factors and develop appropriate detection techniques for the pathogen.

The detection of pathogens is a crucial step in the development and application of suitable disease control methods. A suitable pathogen diagnostic technique needs to be simple, quick, sensitive, specific, and cost-effective in order to be useful for epidemiological investigations and disease control decisions. Several detection techniques are commonly used for bacterial pathogens: biosensor-, immunology-, and nucleic acid-based methods (Law *et al.*, 2015). Nucleic acid-based diagnostic methods have been attracting increasing attention due to their ability to distinguish bacteria that do not exhibit any significant phenotypic differences. Loop-mediated isothermal amplification (LAMP) is a nucleic acid-based method that has emerged as a preferred detection strategy due to its sensitivity, specificity, simplicity, and rapidity (Parida *et al.*, 2008). In contrast to polymerase chain reaction (PCR) technology, in which the reaction is performed with a series of alternating temperature steps, LAMP is an isothermal nucleic acid amplification technique and does not require a thermal cycler, making it more cost-effective. In addition, by using visual detection techniques, such as measuring turbidity caused by the magnesium pyrophosphate precipitate as a byproduct of amplification or observing the fluorescence of intercalating dyes, it is possible to detect a pathogen with the naked eye, reduce assay times, and use simpler equipment. Therefore, LAMP has potential as a simple screening assay in the field.

In the present study, we aimed to (1) isolate *Methylobacterium* strains from rice leaves and confirm whether they are the causal pathogens of leaf bleaching disease in rice, (2) morphologically, biochemically, and genetically characterize the pathogenic agents (*Methylobacterium*), and (3) develop a rapid and sensitive LAMP detection method for pathogenic *Methylobacterium*. The results obtained provide insights into novel rice pathogenic bacteria and a basis for elucidating the mechanisms underlying this novel rice disease, which will contribute to disease control.

## Materials and Methods

### Collection of diseased rice plants and isolation of *Methylobacterium* strains

**Fig. 1.**
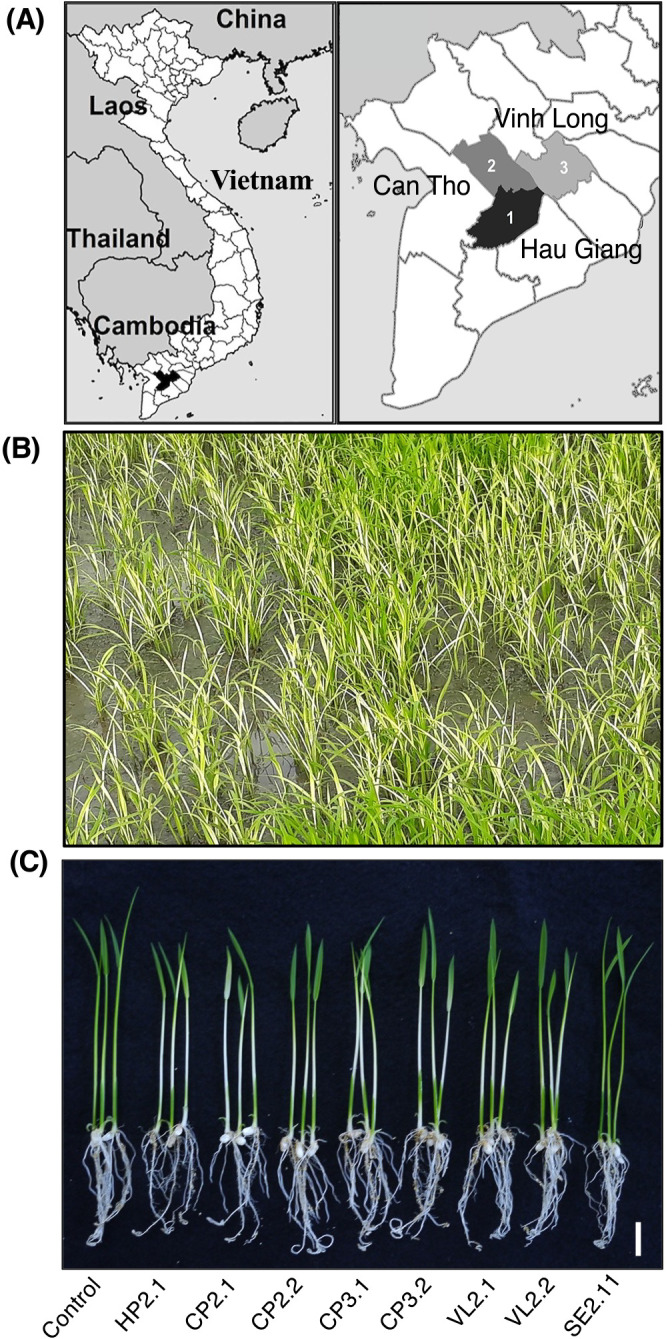
Bleaching symptoms on rice seedlings in the Mekong Delta region of Vietnam. **(A)** Map of sampling sites. Rice plants with leaf bleaching symptoms were collected from 3 provinces (Latitude/Longitude): Hau Giang (9.757898/105.641253), Can Tho (10.027254/105.769806), and Vinh Long (10.254260/105.972298), in the Mekong Delta, Vietnam. **(B)** Rice plants exhibiting bleaching symptoms in Hau Giang in December 2017. **(C)** Rice bleaching symptoms induced by *Methylobacterium* isolates. Rice plants (*Oryza sativa* cv. Nipponbare) were inoculated with *Methylobacterium* isolates HP2.1 (from Hau Giang); CP2.1, CP2.2, CP3.1, and CP3.2 (from Can Tho); VL2.1 andVL2.2 (from Vinh Long); and non-pathogenic *Methylobacterium* strain SE2.11^T^. Control, Non-inoculated plant. Photographed at 7 DAI. Scale bar=1‍ ‍cm.

In December 2017, rice plants (20 days old) with leaf bleaching symptoms appeared in fields in three provinces (Hau Giang, Can Tho, and Vinh Long) in the Mekong Delta of Vietnam (Fig. 1A and B). Five symptomatic plants from at least three different rice fields in each province were collected. The fresh leaves of symptomatic plants were mixed, and 10‍ ‍g of leaf samples was homogenized with 90‍ ‍mL of a sterilized 0.85% NaCl solution and plated onto Murashige and Skoog (MS) medium supplemented with 0.5% (v/v) methanol and 10‍ ‍μg mL^–1^ cycloheximide. Plates were incubated at 28°C for one week. Ten single, well-isolated, pink colonies from each sample were randomly selected and re-streaked to obtain pure cultures. All strains used in the present study are listed in Table S1.

### Pathogenicity tests

Pathogenicity tests on bacterial isolates were conducted using the rice cultivar *Oryza sativa* cv. Nipponbare. Rice seeds were sterilized by soaking in 70% ethanol for 1‍ ‍min and 5% sodium hypochlorite with 0.02% Tween 20 for 10‍ ‍min, followed by washing five times with sterile water. Bacterial isolates were grown in 5‍ ‍mL CMS medium (MS medium with sucrose 30‍ ‍g‍ ‍L^–1^, peptone 2‍ ‍g‍ ‍L^–1^, and tryptone 2‍ ‍g L^–1^) for 4 days. Bacterial cells were harvested by centrifugation at 5,000×*g*, and the pellets obtained were washed twice with sterile water. Cells were diluted to final concentrations of 10^5^ CFU mL^–1^ and 10^7^ CFU mL^–1^ in 10‍ ‍mL of sterile water. Surface-sterilized seeds were soaked for 16 h in the bacterial suspension. Control seeds were treated with sterile distilled water without any bacterial culture. Five inoculated seeds were sown in a 50-mL tube containing 15‍ ‍mL of sterile vermiculite (Hirukon S, Hiruishi Kagaku Kogyo) and watered with 25% MS medium without sucrose solution. Three replicates for each treatment condition were performed in all pathogenicity tests. Plants were grown in a growth chamber at 25°C (8 h dark/16 h light cycle) for 7 days. Bleaching symptoms were considered to exist when the green color of seedling leaves turned white. The chlorophyll and carotenoid contents of the rice seedlings were assessed using the 80% (v/v) acetone extract method described by Arnon (1949). Bacteria were re-isolated from symptomatic plants and compared with the original inoculated bacteria. A whole rice seedling that exhibited bleaching symptoms 7 days after inoculation (DAI) was homogenized with 10‍ ‍mL of a sterilized 0.85% NaCl solution and plated onto MS medium supplemented with 0.5% (v/v) methanol. A single purified colony from each sample was randomly selected and subjected to analyses of morphological, biochemical, and genetic characteristics in order to establish whether the inoculated bacteria and re-isolated bacteria were identical.

### Morphological and biochemical characterization of isolates

The biochemical characterization of isolates was performed following the instructions of the API 20 NE biochemical identification system (BioMérieux) (Table S2). The antibiotic susceptibility test was conducted using CMS medium supplemented with the antibiotics listed in Table S3. Stock solutions of each antibiotic were prepared with suitable solvents, including distilled water (for polymyxin, ampicillin, fosfomycin, spectinomycin, streptomycin, neomycin, kanamycin, cefotaxime, and gentamicin), 70% ethanol (for tetracycline), 95% ethanol (for chloramphenicol), and 100% methanol (for rifampicin) (Table S3).

### Genetic identification and phylogenetic analysis

The genomic DNA of selected isolates was extracted using a Wizard Genomic DNA purification kit (Promega) following the manufacturer’s instructions. The 16S rRNA gene and *atpD* gene were amplified using the primer pairs 16AF/1492R (Frank *et al.*, 2008) and atpDF/atpDR (Gaunt *et al.*, 2001), respectively (Table S4). Regarding each gene sequence, the sequences with the closest identities were searched using the Basic Local Alignment Search Tool (BLAST) in the National Center for Biotechnology Information (NCBI) database. Alignments of the 16S rRNA and *atpD* gene sequences and a phylogenetic analysis were performed using MEGA software ver7.0 (Kumar *et al.*, 2016). Phylogenetic trees were constructed via the neighbor-joining method (Saitou and Nei, 1987; Wilson, 2001).

### Genome sequencing and annotation

Whole-genome shotgun sequencing of the VL1 strain was performed using PacBio and Illumina sequencing technologies. A sequencing library for the PacBio platform was constructed using an Accel-NGS XL Library Kit (Swift Biosciences) in accordance with the manufacturer’s protocol. DNA fragments with sizes ranging between 17 and 50‍ ‍kbp were collected with the BluePippin instrument (Saga Science). The final library was run on a PacBio RSII sequencer with SMRT cell v3 and P6-C4v2 chemistry, generating a total of 166,556 reads (2.34 Gbp). The assembly of PacBio reads was performed with an HGAP3 assembler with the default settings. A paired-end library for the Illumina platform was prepared using a TruSeq DNA PCR-Free Library Prep Kit (Illumina). Fragments of approximately 600 bp were excised from an agarose gel using a Zymoclean Large Fragment DNA Recovery Kit (Zymo Research). The sequencing library was run on an Illumina HiSeq 2500 sequencer with a read length of 250 bp. Illumina read sequences comprising 4,870,746 reads (1.22 Gbp) were used to improve draft sequences by applying Pilon v1.22 and were assembled using Platanus v1.2.4 for the detection and sequence verification of plasmids. The complete genome sequence was automatically annotated using the DDBJ Fast Annotation and Submission Tool (DFAST) (Tanizawa *et al.*, 2018).

### Genome comparisons and ortholog analysis

A circular genome map showing the GC skew and GC content was generated using the CGview server (Grant and Stothard, 2008) with the default parameters. Similarities between strain VL1 and other bacteria were compared using GenomeMatcher (Ohtsubo *et al.*, 2008). The average nucleotide identity (ANI) calculator (www.ezbiocloud.net/tools/ani) was used to calculate ANI values (Yoon *et al.*, 2017) (Table S5). *In silico* DNA-DNA hybridization (DDH) was conducted with the Genome-to-Genome Distance Calculator web service (GGDC 2.1) (Meier-Kolthoff *et al.*, 2013) (Table S5). Secondary metabolite gene clusters were predicted using antiSMASH 5.0, a web-based analysis platform (http://antismash.secondarymetabolites.org) (Blin *et al.*, 2019) (Table S6). The SEED categories of the genome were assigned by applying the Rapid Annotations using Subsystems Technology (RAST) tool (Aziz *et al.*, 2008) (Table S7). Candidate marker sequences in the VL1 genome were identified by comparing the genomes of strain VL1 and other *Methylobacterium* species via BLASTN comparisons with an e-value cut-off of 10^–20^ (Table S8).

### LAMP reaction

To achieve the timely and accurate detection of pathogenic *M. indicum* strains in field or rice samples, a LAMP-based assay was developed.

Searches for the unique marker sequences of pathogenic *M. indicum* strain VL1 were performed under the following criteria: (1) sequences found by an ortholog analysis within the genus *Methylobacterium* to be singleton genes specifically retained only by the VL1 strain; (2) minimum and maximum gene lengths of 500 and 1,200 bp, respectively; (3) no hits in BLASTN searches of the genome sequences of strains SE2.11^T^ and SE3.6; (4) no BLASTN hits in the nr/nt database; and (5) gene sequences with a GC content deviating by less than ±5% from the average chromosomal GC content of 71% (a low GC content indicates horizontal gene transfer). Twenty-two sequences met the above criteria. Furthermore, since the sequence of the plasmid pVL1-2 is highly homologous to a phage sequence, 4 sequences of pVL1-2 were excluded to completely eliminate the possibility of considering a mobile element as a marker sequence for detecting the VL1 strain. Therefore, 18 sequences were selected as candidate marker sequences (Table S8). These unique sequences were searched again for homology using BLAST at the NCBI website. No homologous genes were revealed, which indicated that these 18 sequences were unique to strain VL1. The unique sequences (4 and 6) located on the chromosome of strain VL1 were selected for subsequent steps.

Primers were designed for the LAMP reaction using Primer Explorer V5 software (https://primerexplorer.jp/e/) targeting the unique sequences of pathogenic *Methylobacterium* strains. The primer set consisted of six primers, including two outer primers (F3 and B3), two inner primers (FIP and BIP), and two loop primers (LF and BF). The nucleotide sequences of the primer sets used in the present study are listed in Table S4.

LAMP reactions were performed as previously described (Komatsu *et al.*, 2016), with slight modifications. Briefly, the LAMP reaction mixture contained 25‍ ‍μL of 20‍ ‍mM Tris-HCl (pH 8.8), 8‍ ‍mM MgSO_4_, 10‍ ‍mM (NH_4_)_2_SO_4_, 10‍ ‍mM KCl, 0.1% Tween 20, 0.8 M betaine, each dNTP at 1.4‍ ‍mM, each F3 and B3 primer at 0.2‍ ‍μM, each FIP and BIP primer at 1.6‍ ‍μM, each LF and LB primer at 0.8‍ ‍μM, 1‍ ‍μL of fluorescent detection reagent (Eiken Kagaku), *Bst* DNA polymerase (8 U) (Nippon Gene), and 30‍ ‍ng of the DNA extract as a template. Amplification was performed at 60–67°C for 40–60‍ ‍min, followed by an incubation at 98°C for 10‍ ‍min with Genie II (OptiGene) to detect and monitor fluorescence.

Seven-day-old plants were employed for the LAMP assay using infected rice plants. The root and leaf surfaces of infected rice plants were sampled using a sterilized tip, which was then dipped directly into the LAMP reaction mixture before amplification. Leaf extracts were obtained by crushing 0.05‍ ‍g of symptomatic leaves with 100‍ ‍μL of distilled water in a 1.5-mL sterilized Eppendorf tube using a sterilized microtube pestle (AS ONE), and a 1-μL aliquot of the extract diluted 30 times was used as a template for LAMP.

## Results

### Isolation of pathogenic bacterial strains causing leaf bleaching disease from rice plants grown in the Mekong Delta, Vietnam

Approximately 15 rice plants showing leaf bleaching disease symptoms were collected from the Hau Giang, Vinh Long, and Can Tho provinces (Fig. 1A and B). Thirty pink-pigmented facultative methylotrophic bacteria (PPFMs) were independently isolated on selective medium. All 30 PPFMs were inoculated on rice seeds and examined for their ability to cause bleaching symptoms. Seven isolates produced leaf bleaching on rice plants (Fig. 1C). No significant differences were observed in the onset or severity of disease symptoms among the 7 isolates. The bacteria re-isolated from symptomatic plants exhibited similar morphological, biochemical (by the API test), and genetic (by 16S rRNA gene sequencing) characteristics to the inoculated strains, confirming that these bacteria are the causative agents of bleaching symptoms.

**Fig. 2.**
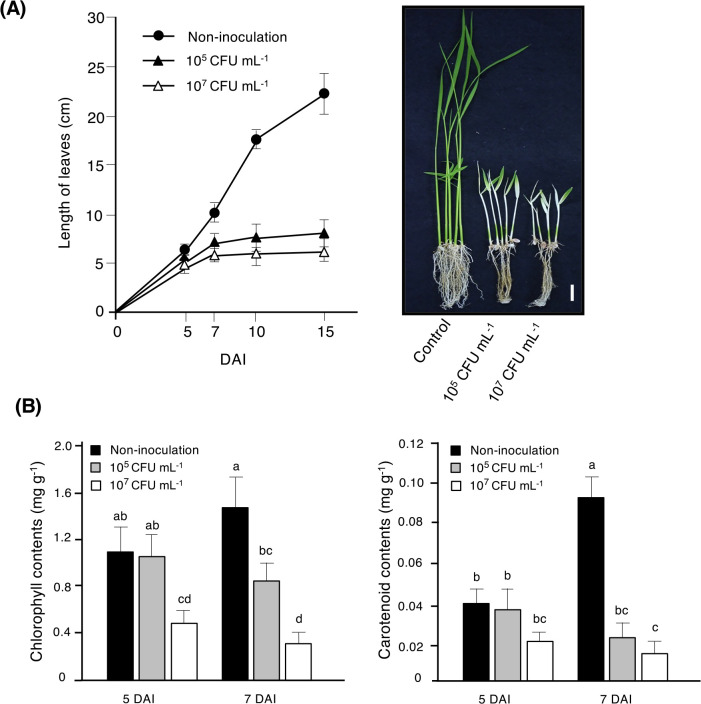
Effects of a pathogenic isolate on the growth of rice seedlings. Rice seeds were inoculated with the *Methylobacterium* isolate CP2.1 at concentrations of 10^5^ CFU mL^–1^ and 10^7^ CFU mL^–1^ and grown until 15 days after inoculation (DAI), as described in the Materials and Methods section. **(A)** The leaf length of rice plants inoculated with CP2.1. A photograph was taken 15 DAI. Scale bar=1‍ ‍cm. **(B)** The chlorophyll and carotenoid contents of rice seedlings were evaluated at 5 and 7 DAI. Values represent the mean±SD. Means followed by different letters are significantly different at the 0.05 level by Tukey’s test with 3 replications. The fresh weight of control plants and 10^5^ CFU mL^–1^- and 10^7^ CFU mL^–1^-inoculated plants were 0.14±0.017 (g), 0.071±0.01 (g), and 0.065±0.01 (g), respectively.

We then investigated the onset and severity of symptoms using different inoculum concentrations. In comparisons with uninoculated plants, the leaf length of rice plants inoculated with the pathogenic strain CP2.1 began to decrease at 5 DAI and continued to decrease thereafter (Fig. 2A). At 15 DAI, inoculated plants were almost completely bleached (Fig. 2A). When higher cell numbers (10^7^ CFU mL^–1^) were applied, leaf bleaching appeared earlier because the leaves of the inoculated rice seedlings began to gradually turn white at approximately 5 DAI. When lower cell numbers (10^5^ CFU mL^–1^) were used, the onset of symptoms was delayed and only became visible at approximately 7 DAI (Fig. S1). The chlorophyll and carotenoid contents of the plants inoculated with lower cell numbers (10^5^ CFU mL^–1^) showed no significant decrease at 5 DAI; however, when higher cell numbers (10^7^ CFU mL^–1^) were used, chlorophyll and carotenoid contents both significantly decreased at 5 DAI. At 7 DAI, lower cell numbers (10^5^ CFU mL^–1^) resulted in significant decreases in chlorophyll and carotenoid contents of 42 and 75%, respectively, from those in uninoculated plants, while the inoculation with higher cell numbers induced a marked decrease, characterized by decreases of 79 and 85%, respectively (Fig. 2B).

### Genetic and biochemical analyses of isolates causing rice bleaching disease

**Fig. 3.**
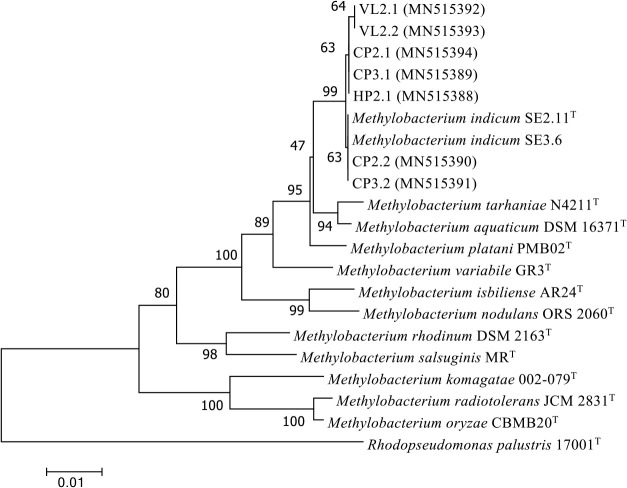
Phylogenetic tree based on 16S rRNA gene sequences of pathogenic *Methylobacterium* isolates. The phylogenetic tree was constructed using approximately 1,400-bp nucleotide sequences of the 16S rRNA gene from 7 *Methylobacterium* isolates and type strains belonging to the genus *Methylobacterium*. Bootstrap values are expressed as percentages based on 1,000 replications. *Methylobacterium* isolates HP2.1 (from Hau Giang); CP2.1, CP2.2, CP3.1, and CP3.2 (from Can Tho); and VL2.1 and VL2.2 (from Vinh Long).

To genetically identify pathogenic isolates, we sequenced their 16S rRNA genes. The 16S rRNA sequences of the isolates (accession numbers MN515388–MN515394) showed 99.25–99.55% identity with that of *M. indicum* strain SE2.11^T^. The phylogenetic tree of 16S rRNA genes showed that these isolates clustered into a single group with *M. indicum* with a high bootstrap score (>99%) (Fig. 3). The *atpD* gene sequences of the isolates (MN809915–MN809921) also indicated that they were the most similar to *M. indicum*, sharing 98.96% sequence identity with *M. indicum* SE2.11^T^ (Fig. S2). These results were consistent with findings on the previously isolated rice bleaching strain *M. indicum* VL1 (previously published as VP43.2; [Lai *et al.*, 2020]).

We then characterized pathogenic isolates through morphological and biochemical analyses. All 7 isolates were pink-pigmented, Gram-negative, rod-shaped, obligate aerobes (Table S2) (Fig. S3 and S4). The oxidase, indole, methyl red, and Voges–Proskauer reactions were negative. Casein and esculin were not hydrolyzed. Neither D-glucose acidification nor L-arginine dihydrolase was observed; however, the following compounds were assimilated: D-glucose, L-arabinose, D-mannose, D-mannitol, N-acetyl-glucosamine, D-maltose, potassium gluconate, adipic acid, malic acid, and trisodium citrate. Differences among some isolates were detected in the L-arginine and aesculin ferric citrate tests (Table S2).

These isolates showed the same pattern of antibiotic susceptibility and were resistant to polymyxin (200‍ ‍μg‍ ‍mL^–1^), ampicillin (200‍ ‍μg‍ ‍mL^–1^), and fosfomycin (200‍ ‍μg‍ ‍mL^–1^), but sensitive to other antibiotics, such as gentamicin (10‍ ‍μg‍ ‍mL^–1^), neomycin (10‍ ‍μg‍ ‍mL^–1^), tetracycline (10‍ ‍μg‍ ‍mL^–1^), kanamycin (10‍ ‍μg mL^–1^), rifampicin (10‍ ‍μg‍ ‍mL^–1^), cefotaxime (10‍ ‍μg‍ ‍mL^–1^), and chloramphenicol (10‍ ‍μg‍ ‍mL^–1^); strain HP2.1 was excluded because it was resistant to spectinomycin and streptomycin (100‍ ‍μg mL^–1^) (Table S3). According to these data, 7 isolates exhibited very similar morphological and biochemical characteristics, which were indistinguishable from the previously isolated bleaching strain *M. indicum* VL1 and the non-pathogenic *M. indicum* SE 2.11^T^ and SE3.6 strains.

### Whole-genome sequence of pathogenic *M. indicum* strain VL1

**Fig. 4.**
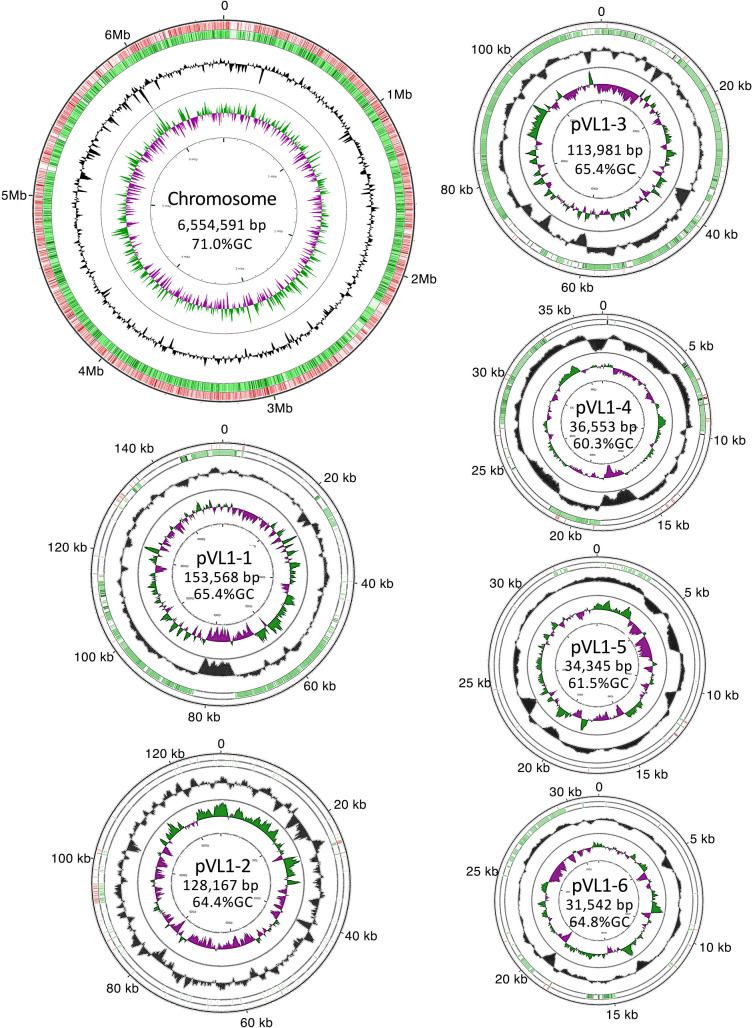
The genome structure of *Methylobacterium indicum* VL1. Circular representation of the chromosome and 6 plasmids of VL1. The outermost and second circles represent BLASTN comparisons with *M. extorquens* AM1 (Accession number NC_012808) and *M. indicum* SE2.11^T^ (Accession number JTHF00000000), respectively (e-value <10^–10^). The innermost and second-innermost circles show the GC skew and GC content, respectively. The GC content circle shows the deviation from the average GC content of the entire sequence (a higher than average GC content is represented in green, while a lower than average content is shown in purple).

Based on the genetic and biochemical characteristics observed, the pathogenic isolates were identified as *M. indicum* and were indistinguishable from the non-pathogenic *M. indicum* SE 2.11^T^ and SE3.6 strains. To gain insights into the molecular basis of the novel pathogenic *Methylobacterium*, we sequenced the whole genome of pathogenic *M. indicum* strain VLl. The length of the whole genome of VL1 was 7.05‍ ‍Mbp, and the GC content was 70.5% (Table 1). The genome size and GC content of VL1 were similar to those of *M. indicum* strains SE2.11^T^ and SE3.6, respectively (Table 1). However, while the genomes of SE2.11^T^ and SE3.6 consisted of a single chromosome, that of VL1 comprised a chromosome (6,554,591 bp) and 6 plasmids (153,568, 128,167, 113,981, 36,553, 34,345, and 31,542 bp) (Fig. 4). BLASTN comparisons revealed that the chromosome showed overall homology to those of *M. indicum* strain SE2.11^T^ and *Methylobacterium extorquens* AM1, whereas the plasmids showed only limited homology with *M. indicum* strain SE2.11^T^ (Fig. 4).

*In silico* DDH values between strain VL1 and the reference strains were calculated with GGDC 2.1. The DDH value of VL1 with *M. indicum* strains SE2.11^T^ and SE3.6 was 86.0% (Table S5) and was higher than the DDH threshold of 70% established for prokaryotic species delineation (Auch *et al.*, 2010). In contrast, the DDH values of VL1 with other *Methylobacterium* species varied between 16.9% (*Methylobacterium* sp. 17SD2) and 52.8% (*Methylobacterium aquaticum* MA-22A), which were below the 70% DDH threshold, suggesting that they are different species. In addition, the ANI was calculated as an overall genome relatedness index in pairwise comparisons between *M. indicum* strain VL1 and other *Methylobacterium* strains (Richter and Rossello-Mora, 2009; de Lajudie *et al.*, 2019). The highest ANI values, obtained from comparisons of VL1 versus strains SE2.11^T^ (98.19%) and SE3.6 (98.18%), were higher than the 95% ANI threshold established for prokaryotic species delineation, suggesting that these strains belonged to the same species (Table S5). On the other hand, the ANI values of VL1 with other strains ranged between 77.29% (*Methylobacterium* sp. XJLW) and 91.85% (*Methylobacterium aquaticum* MA-22A), which were below the threshold. Taken together with the 16S rRNA and *atpD* gene phylogenetic analyses, these results indicated that VL1 belongs to *M. indicum*.

### Genome features of pathogenic *M. indicum* strain VL1

The VL1 genome includes 6,599 putative coding sequences (CDSs) and encodes 36 rRNAs and 114 tRNAs. Among the 6,599 CDSs, 3,260 (48%) were classified as hypothetical, while 1,426 (22%) were assigned to various RAST categories according to subsystems (Table S6). A major fraction of annotated genes was related to amino acids and their derivatives (387), carbohydrate metabolism (240), protein metabolism (201), and cofactors, vitamins, prosthetic groups, and pigments associated with metabolism (188).

Due to the bleaching-inducing traits of strain VL1, its potential to produce secondary metabolites acting as phytotoxins was analyzed. The GenBank file of the annotated genome was employed as the input for the online pipeline antiSMASH 5.0, and the software then automatically assigned 11 putative gene clusters involved in the biosynthesis of different natural products, including Terpene, T1PKS, homoserine lactone (hserlactone), NAPPA, and redox cofactor clusters (Table S7). No gene clusters involved in phytotoxin biosynthesis or virulence were identified. These results were consistent with those obtained on the RAST subsystems (Table S6). Therefore, the bleaching disease caused by VL1 may involve a novel mechanism that is different from those previously reported.

### Development of a LAMP assay for the detection of pathogenic *Methylobacterium* strains

To perform a more comprehensive survey of pathogenic *Methylobacterium* strains in rice fields, a rapid detection system based on the LAMP method was established. Primers were designed from the 18 unique sequences of VL1 using Primer Explorer V5 (Table S8). Two primer sets, designated as Chr1 and Chr2, which targeted sequences 4 and 6 in the chromosome, respectively, were selected (Table S4). To select a LAMP primer set, we initially performed conventional PCR using pathogenic and non-pathogenic *Methylobacterium* strains. The two primer pairs (Chr1-F3/B3) and (Chr2-F3/B3) belonging to the primer sets (Chr1 and Chr2) successfully amplified fragments from 10 pathogenic *Methylobacterium* strains, while no fragments were amplified from the non-pathogenic *Methylobacterium* strain SE2.11^T^ using PCR reactions (Fig. S5). These results indicated the potential of these unique sequences for use in distinguishing between pathogenic and non-pathogenic *Methylobacterium* strains.

**Fig. 5.**
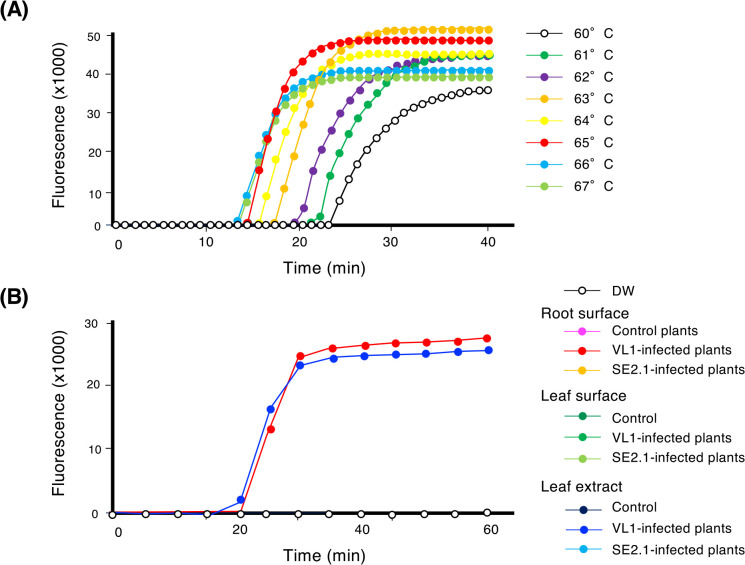
Detection of a pathogenic *Methylobacterium* strain using loop-mediated isothermal amplification (LAMP). **(A)** LAMP assay using the Chr1 primer set and 30‍ ‍ng of extracted genomic DNA of *Methylobacterium* strain VL1. LAMP reactions were performed at different temperatures between 60 and 67°C for 40‍ ‍min. The time interval between symbols was 1‍ ‍min. **(B)** LAMP assay using the Chr1 primer set and rice plants infected with *Methylobacterium* strains. The leaf surface, root surface, and leaf extract were used for LAMP reactions performed at 65°C for 60‍ ‍min. Similar results were obtained in three independent experiments. The time interval between each symbol was 5‍ ‍min.

LAMP reactions successfully detected VL1 at 60–70°C (Fig. 5A). Considering the detection time, final fluorescence intensity, and optimum DNA polymerase temperature (55–65°C), we performed subsequent LAMP assays at 65°C. The specificity of the LAMP assay was tested using the pathogenic and non-pathogenic *Methylobacterium* strains (Table S9). The LAMP assay developed successfully detected all three pathogenic *Methylobacterium* isolates, while signal intensity was under the detection level for non-pathogenic *Methylobacterium* strains even after a 60-min incubation. These results suggested that the established LAMP assay specifically distinguishes pathogenic strains from non-pathogenic *Methylobacterium* strains.

The detectable concentration of the target based on the established LAMP assay was also examined using a cell suspension of strain VL1. The results obtained showed that the LAMP assay detected strain VL1 in as few as 50 cells reaction^–1^ using the Chr1 primer set and 100 cells reaction^–1^ using the Chr2 primer set (Table S10). The Chr1 primer set was selected for further experiments due to its higher sensitivity of detection than the Chr2 primer set.

We also investigated whether the developed LAMP assay was able to directly detect pathogenic *Methylobacterium* strains from infected plants. The root surfaces, leaf surfaces, and leaf extracts of 7-day-old VL1-infected rice seedlings were used as samples for LAMP reactions. The results obtained indicated that the LAMP assay using the Chr1 primer set successfully detected pathogenic *Methylobacterium* strains from root surfaces and leaf extracts, but not from the leaf surfaces of infected rice plants, while no positive amplification was observed from the uninoculated plants (Fig. 5B). These results indicate the potential of the developed LAMP assay to detect pathogenic *Methylobacterium* strains in field samples.

## Discussion

The emergence of rice bleaching disease represents a threat to farmers in the Mekong Delta, Vietnam (Khuyen Nong, 2010). The bleaching symptoms observed significantly differ from those of other known rice diseases. Cold damage symptoms in rice were previously described as white bands appearing on the leaf blade during exposure to cold temperatures (da Cruz *et al.*, 2013). Bacterial leaf blight caused by *Xanthomonas oryzae* is characterized by wavy elongated lesions. These lesions initially appear as small water-soaked stripes from the leaf tips and spread along the leaf margins, which eventually become white-grey (Sharma *et al.*, 2017). In contrast, in the bleaching disease investigated herein, bleaching symptoms were recognized when white areas appeared on the leaves, which then slowly spread to cover the entire rice plant. When this symptom developed, infected rice plants grew slowly and eventually died as a result of heavily bleached leaves. However, the agents causing these symptoms have not yet been identified. Although farmers are currently implementing measures based on their own experience to protect rice plants from this disease, the effects of these measures are not stable (Khuyen Nong, 2010). Therefore, the identification of the agents causing leaf bleaching disease and development of control measures based on the characteristics of the causative agents are very important steps for controlling this disease. In the present study, we isolated and characterized causative agents from diseased rice in the Mekong Delta in Vietnam. The inoculation of 7 *Methylobacterium* isolates caused bleaching symptoms on rice seedlings, and these bacteria were reisolated from the diseased plants and identified as the inoculated strains, thereby fulfilling Koch’s postulates.

When rice was infected with the pathogenic *Methylobacterium* strains, the green color of the rice leaves was gradually bleached and the leaves turned white. These characteristics are consistent with the decreases detected in chlorophyll and carotenoid contents (Fig. 2B). In many plant-pathogen interactions, when phytopathogens invade host plants, they degrade plant cell walls and lipid membranes, which facilitates the entry of the pathogen into the host plant (Ghorbani *et al.*, 2005). Phytopathogens may also produce various phytotoxic secondary metabolites that interfere with plant metabolism, which may directly or indirectly induce gene expression, eventually leading to plant cell death (Stergiopoulos *et al.*, 2013). In the present study, although the pathogenic *Methylobacterium* strains caused some degree of photosynthetic pigment loss, no necrosis or lesions were observed on the surface of the leaves or roots of the infected plants. Therefore, these pathogens may produce a phytotoxic compound that directly or indirectly interferes with photosynthesis-related functions, leading to growth retardation in rice plants. The identification of the responsible phytotoxic compound will provide insights into the molecular mechanisms underlying the bleaching symptoms of pathogenic *Methylobacterium* strains.

Analyses of 16S rRNA and *atpD* gene sequences revealed that all of the pathogenic isolates obtained belonged to *M. indicum*. *Methylobacterium* species have been associated with more than 70 plant species, including endophytes, epiphytes, or endosymbionts (Sy *et al.*, 2001; Lacava *et al.*, 2004; Omer *et al.*, 2004). Lacava *et al.* (2004) reported that *Methylobacterium* species are important citrus endophytes that directly interact with the pathogen *Xylella fastidiosa*. They promote plant growth and induce defense responses against pathogens in plants such as rice and groundnuts (Madhaiyan *et al.*, 2006). Other studies demonstrated that *Methylobacterium* species possess genes related to nodulation and nitrogen fixation and that they establish nitrogen-fixing symbioses by nodulating legume roots (Sy *et al.*, 2001). However, prior to our findings on these *M. indicum* strains isolated from Vietnamese rice fields, *Methylobacterium* species had never been reported to cause disease symptoms in plants. The identity and origin of the pathogenic genes in pathogenic *Methylobacterium* strains warrant further study to elucidate how this pathogen evolved to acquire pathogenicity.

The whole genome of pathogenic *Methylobacterium* strain VL1 was sequenced. *In silico* DDH and ANI results revealed that the strain belonged to *M. indicum*. Chaudhry *et al.* (2016) initially reported *M. indicum* strains (SE2.11^T^ and SE3.6) isolated from surface-sterilized rice seeds in India. These strains did not cause leaf bleaching symptoms, but were similar to the identified pathogenic strains in terms of their morphological and biochemical characteristics and antibiotic susceptibility. *Methylobacterium* species have been detected in various environments, including soil, water, the leaf surface, nodules, seed grains, and air environments (Wellner *et al.*, 2012; Madhaiyan *et al.*, 2015). Some *Methylobacterium* strains possess the ability to be transmitted mainly through seeds (Madhaiyan *et al.*, 2015). Pathogenic *M. indicum* strains may have been transmitted between India, Vietnam, and other countries through rice import and export activities. However, their pathogenic characteristics have changed in various ways that may be related to evolutionary adaptation under different environmental conditions (Nayak and Marx, 2014; Engelhardt and Shakhnovich, 2019). The RAST analysis revealed that 10 genes related to phages, prophages, transposable elements, and plasmids were distributed among the chromosome and plasmid 2 of VL1 (pVL1-2) (Table S6). The horizontal transfer of transposable elements plays a key role in the evolution of the prokaryote genome. Some characteristics related to the antibiotic resistance, virulence, and pathogenicity of this strain may be modified by horizontally transferred genes, the transfer of which was mediated by other mobile genetic elements, such as phages and plasmids (Leclercq *et al.*, 2012). However, we did not identify genes related to virulence, disease, or defense that were assigned to the genome of VL1 by RAST. These results suggest that the genes responsible for phytotoxin production have yet to be identified. Two putative gene clusters related to homoserine lactone (hserlactone) were detected in the VL1 strain genome, but not in those of strains SE2.11^T^ and SE3.6 by the antiSMASH 5.0 analysis. In many Gram-negative bacteria, including members of the genus *Methylobacterium*, N-acyl-L-homoserine lactone (AHL) acts as a quorum-sensing signal (Madhaiyan *et al.*, 2007). AHL-mediated quorum sensing regulates the expression of numerous genes involved in various processes, including biofilm formation, pigment production, signal turnover, luminescence, antibiotic production, swarming, and virulence (Ng and Bassler, 2009; Kimura, 2014). Strain VL1 may use the quorum-sensing system to regulate some genes related to its bleaching ability. Further studies are needed to identify the pathogenic genes and elucidate the physiological mechanisms underlying this novel bleaching disease of rice.

The present study successfully developed a LAMP assay for the rapid and sensitive detection of pathogenic *Methylobacterium* strains. This allows the pathogen to be detected without relying on complex and time-consuming steps, including bacterial isolation, biochemical tests, pathogenicity tests, and molecular identification. Despite their similarities, the LAMP assay showed the ability to differentiate between pathogenic and non-pathogenic strains. In addition, the established LAMP assay showed a detection limit of 50 cells reaction^–1^ (Table S10), which is similar to the limits of previously reported LAMP-based detection methods for plant pathogens, such as *Ralstonia solanacearum* (Lenarčič *et al.*, 2014), *Erwinia amylovora* (Bühlmann *et al.*, 2013), and *Xanthomanas albilineans* (Duarte Dias *et al.*, 2018). In these studies, at least 30–60‍ ‍min was required for the preparation of bacterial cells or DNA extraction. In our LAMP assay, reactions are performed directly in bacterial cells without the requirement for DNA extraction or any additional treatments, thereby reducing costs and reaction times. This quick and simple procedure also reduces the risk of cross-contamination by aerosols, which may produce false positives (Komatsu *et al.*, 2016). In addition, the developed LAMP assay successfully detected pathogenic *Methylobacterium* strains from the root surfaces of infected rice plants, while no positive amplification from uninoculated plants was observed (Fig. 5B). However, the LAMP assay was unable to detect the pathogen from the leaf surfaces of infected plants, which may have been due to the lower number of bacteria colonizing the leaf surface than the root surface. An improved method for leaf surface sampling was established by crushing leaf samples in distilled water, followed by the use of homogenized products as a template for the LAMP reaction. With this method, amplification was observed from leaf extract samples, but not from uninoculated plants. In real rice fields, the sampling of leaves is easier than that of roots, and the alternative method may benefit from improvements to the sampling technique. The further validation and optimization of the developed LAMP assay using various types of samples from rice fields (*e.g.*, soils, paddy water, and rice seeds) are needed to achieve high reliability in the application of this method in future disease surveys. The development of a rapid and simple detection method is important as the first step for elucidating the pathogen transmission route and achieving disease control.

### Nucleotide sequence accession numbers

The DNA sequences of isolates were deposited in the DNA Data Bank of Japan (DDBJ) under accession numbers MN515388–MN515394 (16S rRNA) and MN809915–MN809921 (*atpD*). The sequences of the chromosome and plasmids of strain VL1 are available in the DDBJ/GenBank/EMBL database (accession numbers AP024145.1–AP024151.1). The raw reads of the VL1 genome are available in the DDBJ Sequence Read Archive (SRA) under accession number DRR259324. The whole-genome shotgun (WGS) project of SE2.11 and SE3.6 have the project accession numbers JTHG00000000 and JTHG01000000, respectively.

## Citation

Lai, K., Nguyen, N. T., Yasuda, M., Dastogeer, K. M. G., Toyoda, A., Higashi, K., et al. (2021) Leaf Bleaching in Rice: A New Disease in Vietnam Caused by *Methylobacterium indicum*, Its Genomic Characterization and the Development of a Suitable Detection Technique. *Microbes Environ ***36**: ME21035.

https://doi.org/10.1264/jsme2.ME21035

## Supplementary Material

Supplementary Material

## Figures and Tables

**Table 1.** General genome features of *Methylobacterium indicum* VL1 and close relatives.

	***M. indicum*** ** VL1**	**SE2.11^T^**	**SE3.6**
**Genome size (bp)**	7,052,747	6,945,341	6,930,824
**G+C content (%)**	70.5	69.9	70.3
**No. of genes**	6,599	6,374	6,404
**No. of CDSs**	6,599	5,645	5,677
**rRNA genes**	36	10	5
**tRNA genes**	114	54	64
**Plasmids**	6	0	0
**Accession no.**	AP024145–AP024151	JTHF01000000	JTHG01000000
