# Supplementary material for: Leaf Bleaching in Rice: A New Disease in Vietnam Caused by Methylobacterium indicum, Its Genomic Characterization and the Development of a Suitable Detection Technique
Source: Microbes Environ. 2021 Nov 3;36(4):ME21035. doi: 10.1264/jsme2.ME21035 (PMC8674445; doi:10.1264/jsme2.ME21035)
Supplement: Supplementary file 1 — Supplementary Material [file 36_21035_s1.pdf]

Table S1. Bacterial strains used in this study

Strains	Species	Characteristics[a]	References or sources
HP2.1	*M. indicum*	Pathogen	This study
CP2.1	*M. indicum*	Pathogen	This study
CP2.2	*M. indicum*	Pathogen	This study
CP3.1	*M. indicum*	Pathogen	This study
CP3.2	*M. indicum*	Pathogen	This study
VL2.1	*M. indicum*	Pathogen	This study
VL2.2	*M. indicum*	Pathogen	This study
VL1	*M. indicum*	Pathogen	Khoa *et al*., 2020
DP28.3	*M. indicum*	Pathogen	Khoa *et al*., 2020
DP28.4	*M. indicum*	Pathogen	Khoa *et al*., 2020
CP40.4	*M. rhodium*	Nonpathogen	Khoa *et al*., 2020
CP10.4	*M. komagatae*	Nonpathogen	Khoa *et al*., 2020
VR43.1	*M. oryzea*	Nonpathogen	Khoa *et al*., 2020
TP31.4	*M. salsuginis*	Nonpathogen	Khoa *et al*., 2020
TP33.2	*M. radiotolerans*	Nonpathogen	Khoa *et al*., 2020
SE2.11[T]	*M. indicum*	Nonpathogen	Chaudhry *et al*., 2016
SE3.6	*M. indicum*	Nonpathogen	Chaudhry *et al*., 2016

[a]Pathogen; the strain causing bleaching symptom on rice seedlings.

Table S2. Morphological and biochemical characteristics of pathogenic isolates

Characteristics	1	2	3	4	5	6	7	8	9	10	11	12
Pigmentation	Pink	Pink	Pink	Pink	Pink	Pink	Pink	Pink	Pink	Pink	Pink	Pink
Gram	-	-	-	-	-	-	-	-	-	-	-	-
Shape	Rod	Rod	Rod	Rod	Rod	Rod	Rod	Rod	Rod	Rod	Rod	Rod
L-tryptophane	-	-	-	-	-	-	-	-	-	-	-	-
D-glucose	-	-	-	-	-	-	-	-	-	-	-	-
L-arginine	-	-	-	+	-	-	-	-	-	-	-	-
Urea	+	+	+	+	+	+	+	+	+	+	+	+
Esculin Ferric citrate	-	+	-	-	-	-	-	-	-	-	-	-
Gelatin (bovince origin)	-	-	-	-	-	-	-	-	-	-	-	-
4-nitrophenyl-D galactopyranoside	-	-	-	-	-	-	-	-	-	-	-	-
D-glucose	++	++	++	+	+	++	++	++	+	+	+	+
L-arabinose	++	++	++	++	++	++	++	++	++	++	++	++
D-mannose	+	++	+	+	+	+	+	+	+	+	+	+
D-manitol	+	++	+	+	++	+	++	++	+	++	+	+
N-acetyl-glucosamine	+	+	+	+	+	+	+	+	+	+	+	+
D-maltose	+	+	+	+	+	+	+	+	+	+	+	+
Potassium glucomate	++	++	++	++	++	++	++	++	++	++	++	++
Capric acid	-	-	-	-	-	-	-	-	-	-	-	-
Adipic acid	++	++	++	+	++	+	++	++	+	+	+	++
Malic acid	++	++	++	++	++	++	++	++	+	+	+	++
Tridium citrate	++	++	++	+	++	++	++	++	+	+	+	++
Phenylacetic acid	-	-	-	-	-	-	-	-	-	-	-	-

1-10 (pathogenic strains) and 11-12 (nonpathogenic strains). 1. HP2.1; 2, CP2.1; 3. CP2.2; 4. CP3.1; 5. CP3.2; 6. VL2.1; 7. VL2.2; 8. VL1; 9. DP28.3; 10. DP28.4; 11. SE2.11; 12. SE3.6. ++, strong positive; +, weak positive; -, negative.

Table S3. Antibiotic susceptibility of pathogenic isolates

Antibiotics	Concentration (ug/ml)	**1**	**2**	**3**	**4**	**5**	**6**	**7**	**8**	**9**	**10**	**11**	**12**
Polymyxin	200	++	++	++	++	++	++	++	++	++	++	++	++
Ampicillin	200	++	++	++	++	++	++	++	++	++	++	++	++
Fosfomycin	200	+	+	+	+	+	+	+	+	+	+	+	+
	100	++	++	++	++	++	++	++	++	++	++	++	++
	50	++	++	++	++	++	++	++	++	++	++	++	++
	10	++	++	++	++	++	++	++	++	++	++	++	++
Spectinomycin	200	-	-	-	-	-	-	-	-	-	-	-	-
	100	+	-	-	-	-	-	-	-	-	-	-	-
	50	++	+	+	+	+	+	+	+	+	+	+	+
	10	++	++	++	++	++	++	++	++	++	++	++	++
Streptomycin	200	-	-	-	-	-	-	-	-	-	-	-	-
	100	+	-	-	-	-	-	-	-	-	-	-	-
	50	++	+	+	+	+	+	+	+	+	+	+	+
	10	++	++	++	++	++	++	++	++	++	++	++	++
Neomycin	10	-	-	-	-	-	-	-	-	-	-	-	-
Tetracyclin	10	-	-	-	-	-	-	-	-	-	-	-	-
Kanamycin	10	-	-	-	-	-	-	-	-	-	-	-	-
Rifampicin	10	-	-	-	-	-	-	-	-	-	-	-	-
Cefotaxime	10	-	-	-	-	-	-	-	-	-	-	-	-
Chloramphenicol	10	-	-	-	-	-	-	-	-	-	-	-	-
Gentamicin	10	-	-	-	-	-	-	-	-	-	-	-	-

1-10 (pathogenic strains) and 11-12 (nonpathogenic strains). 1. HP2.1; 2, CP2.1; 3. CP2.2; 4. CP3.1; 5. CP3.2; 6. VL2.1; 7. VL2.2; 8. VL1; 9. DP28.3; 10. DP28.4; 11. SE2.11; 12. SE3.6. ++, strong resistance; +, weak resistance; -, sensitive.

Table S4. DNA oligonucleotides used in this study

Oligonucleotides	Sequences (5′ - 3′)	Usage	Sources
16AF	AACTGAAGAGTTTGATCMTGGCTCAG	Amplify 16S rRNA gene	Frank *et al*., 2008
1492R	TACGGYTACCTTGTTACGACTT		
AtpDF	SCTGGGSCGYATCMTGAACGT	Amplify *atpD* gene	Gaunt *et al*., 2001
AtpDR	GCCGACACTTCCGAACCNGCCTG		
**Chr1 primer set**		LAMP reaction target unique sequence 4 located on chromosome of strain VL1	This study
Chr1-F3*	CCGGGACCTACGAGATCC		
Chr1-B3*	GGATGTAGTCCGTCTTCTGC		
Chr1-FIP	TCTCGACGTCACCGAACGTCGTACGGCGAGGTGCTCTA		
Chr1-BIP	GGCCAACCTCAAGGTCACCTCGCCTTCATGGTGAACTC		
Chr1-LF	CCCGAGTAGTTCAGGAGCTTG		
Chr1-LB	CCAGGAAGCAAAGGTACGCCAA		
**Chr2 primer set**		LAMP reaction target unique sequence 6 located on chromosome of strain VL1	This study
Chr2-F3*	ACAAACGCGCCGAGA		
Chr2-B3*	CCTGGTGTCAACGAGGT		
Chr2-FIP	TTCGGCCAGGCTCACCCTGACGGTGACCGACGATGA		
Chr2-BIP	GATCCGCGTCAAGGTCGTGAGGGCGTGAAGCAC		
Chr2-LF	CCGCGCATCTCCTGGAT		
Chr2-LB	CGACGGAACCTACTCTATCGG		

*used for PCR amplification of the target sequence 4 (225 bp) and sequence 6 (243 bp) of pathogenic *Methylobacterium* strains.

Table S5. The comparison of genome sequences of *M. indicum* strain VL1 with *Methylobacterium* species.

**Reference strain (Accesion no.)**	**In silico DDH values**	**ANI (%)**
Methylobacterium indicum SE2.11^T (JTHF00000000.1)	86%	98.19
Methylobacterium indicum SE3.6 (JTHG00000000.1)	86%	98.18
Methylobacterium aquaticum MA-22A (AP014704.1)	52.8%	91.84
Methylobacterium aquaticum DSM 16371^T (LABX00000000.1)	44.9%	87.53
Methylobacterium sp. 17Sr1-28 (CP029553.1)	41.5%	88.24
Methylobacterium sp. 17Sr1-1 (CP029552.1)	40.7%	88.06
Methylobacterium nodulans ORS 2060^T (CP001349.1)	27.6%	80.70
Methylobacterium sp. 4-46 (CP000943.1)	27.5%	80.81
Methylobacterium sp. 17Sr1-43 (CP029551.1)	24.42%	77.83
Methylobacterium radiotolerans JCM 2831 (NC010505.1)	24.2%	77.59
Methylobacterium sp. XJLW (CP016429.1)	23.9%	77.29
Methylobacterium oryzae CBMB20^T (CP003811.1)	23.8%	77.38
Methylobacterium phyllosphaerae CBMB27 (CP015367.1)	23.8%	77.33
Methylobacterium sp. DM1 (CP029174.1)	23.7%	77.49
Methylobacterium sp. AMS5 (CP006992.1)	23.7%	77.37
Methylobacterium extorquens AM1 (CP001001.1)	22%	77.37
Methylobacterium radiotolerans DM4 (FP103042.2)	21.9%	77.34
Methylobacterium sp. 17SD2 (CP029550.1)	16.9%	77.81

Table S6. Summary of subsystems identified by RAST of all annotated CDS in *M. indicum* strain VL1.

**Subsystem**	**Predicted genes***
Cofactors, Vitamin, Prosthetici groups, Pigments	188
Cell wall and Capsule	27
Virulence, Disease, Denfense, Toxins and Superantigens	0
Resistance to antibiotics and toxic compounds	19
Invassion, intracellular resistance	13
Potassium metabolism	9
Photosynthesis	10
Miscellaneoous	26
Phages, Prophages, Transposable elements, Plasmids	10
Membrane transport	61
Iron acquisition and metabolism	8
RNA metabolism	37
Nucleosides and Nucleotides	90
Protein metabolism	201
Cell division and Cell cycle	2
Motility and Chemotaxis	95
Regulation and Cell signaling	41
Secondary metabolism	5
DNA metabolism	86
Fatty acids, Lipids and Isoprenoids	90
Nitrogen metabolism	17
Dormancy and sporulation	1
Respiration	137
Stress response	70
Metabolism of aromatic compounds	65
Amino acids and Derivaties	387
Sulfur metabolism	21
Phosphorus metabolism	26
Carbohydrates	240

*Total protein coding genes as per annotated genome

Table S7. List of predicted gene clusters for secondary metbolite biosynthetis in *M. indicum* strain VL1.

Cluster type	Strain VL1			Strain SE2.11^T	Strain SE3.6
	Most similar known cluster	Location	Similarity		
Terpene	Carotenoids	Chromosome (1018391-1038982)	100%	+	+
T1PKS	nd	Chromosome (2983581-3031038)		+	+
Terpene	nd	Chromosome (3274988-3295023)		+	+
Terpene	nd	Chromosome (3709482-3730165)		+	+
Hserlactone	nd	Chromosome (4145369-4165635)		+	+
Hserlactone	nd	Chromosome (4449927-4469639)		+	+
NAPAA	Chejuenolide A/B	Chromosome (4723337-4757434)	7%	+	+
Redox-cofactor	nd	Chromosome (5377026-5399320)		+	+
Terpene	nd	Chromosome (6001196-6026346)		+	+
Hserlactone	nd	pVL1-1 (105689-126483)		-	-
Hserlactone	nd	pVL1-3 (147-20803)		-	-

nd, not determined; +, same gene cluster was detected; -, no same cluster was detected.

Table S8. The unique sequences in *M. indicum* VL1 genome compared with other *Methylobacterium* strains

Region		Location	Length (bp)
Chromosome	Sequence 1	50800-51879	1080
	Sequence 2	1976371-1977171	801
	Sequence 3	2198767-2199366	600
	Sequence 4*	2224985-2225797	813
	Sequence 5	2225836-2226456	621
	Sequence 6*	2351338-2351904	567
	Sequence 7	2352080-2353243	1164
	Sequence 8	6269032-6269574	543
Plasmid 2 (pVL1-2)	Sequence 1	29907-30482	576
	Sequence 2	32390-33556	1167
	Sequence 3	109929-110534	606
Plasmid 3 (pVL1-3)	Sequence 1	3005-3628	624
	Sequence 2	55686-56258	573
	Sequence 3	71802-72566	765
	Sequence 4	99184-99972	789
	Sequence 5	105406-106020	615
	Sequence 6	109929-110534	606
Plasmid 6 (pVL1-6)	Sequence 1	16160-16681	522

"*", these unique sequences were used as the targets of LAMP reactions establised.

Table S9. The LAMP specificity test.

Bacterial strain	Species	Characteristics	LAMP reaction results	
			Chr1 primer set	Chr2 primer set
VL1	*M. indicum*	Pathogen	+	+
DP28.3	*M. indicum*	Pathogen	+	+
DP28.4	*M. indicum*	Pathogen	+	+
SE2.11	*M. indicum*	Nonpathogen	-	-
SE3.6	*M. indicum*	Nonpathogen	-	-
CP40.4	*M. rhodium*	Nonpathogen	-	-
CP10.4	*M. komagatae*	Nonpathogen	-	-
VR43.1	*M. oryzea*	Nonpathogen	-	-
TP31.4	*M. salsuginis*	Nonpathogen	-	-
TP33.2	*M. radiotolerans*	Nonpathogen	-	-

LAMP, loop-mediated isothermal amplification. Similar results were obtained in three independent experiments.

Pathogen indicates the strain causing bleaching symptom on rice seedlings, nonpathogen indicates the strain could not cause bleaching symptom on rice seedlings.

+, positive reaction; -, negative reaction.

Table S10. The sensitivity LAMP assay developed in this study.

Cells/reaction	LAMP reaction results					
	Chr1 primer set			Chr2 primer set		
10000	+	+	+	+	+	+
1000	+	+	+	+	+	+
100	+	+	+	+	+	+
50	+	+	+	+	-	-
10	+	-	-	+	-	-

LAMP, loop-mediated isothermal amplification. The LAMP reactions were repeated three times for each concentration of pathogenic bacteria (cell/reaction).

+, positive; -, negative.

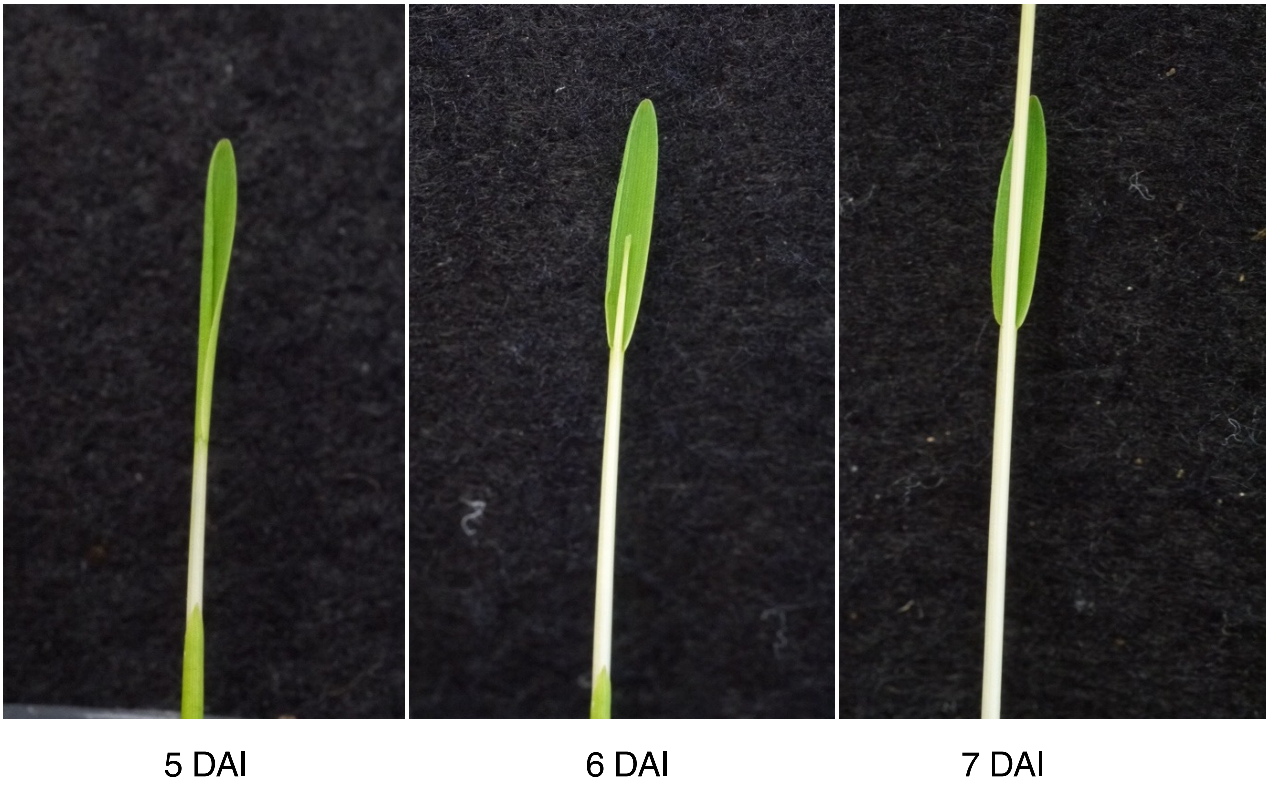

Figure S1. Effect of a pathogenic isolate on the growth of the rice seedlings. Rice seeds were inoculated with a *Methylobacterium* isolate CP2.1 at the concentration of 10^5 CFU/ml and grown as described in materials and methods. The white color of the leaves appeared around 5 days after inoculation (DAI). Plants were photographed at 5 - 7 DAI.

(Lai *et al*., 2021)

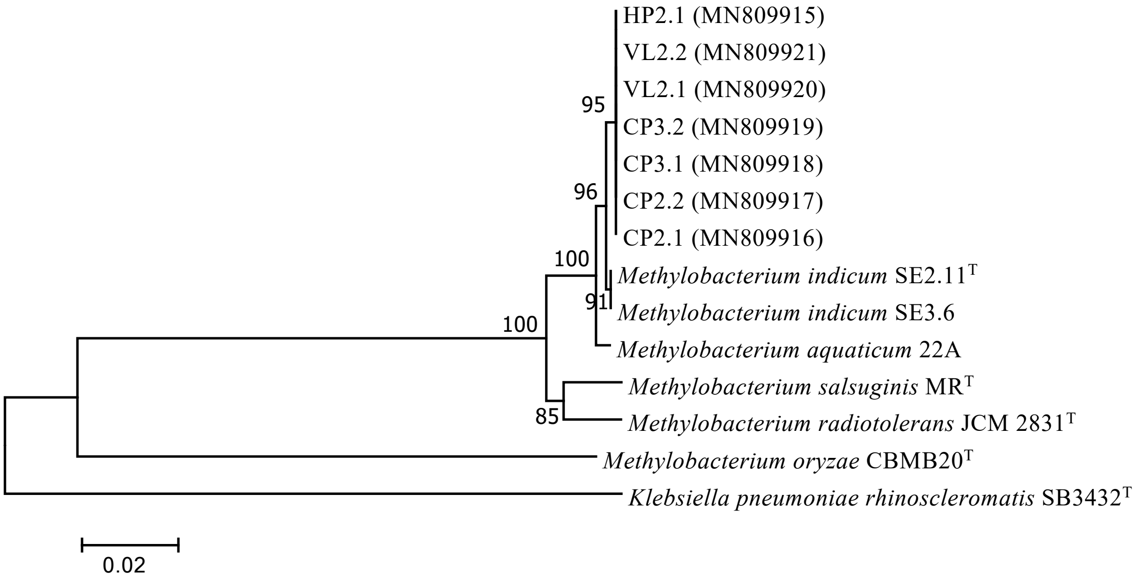

Figure S2. Phylogenetic tree based on the *atpD* gene sequences of the pathogenic *Methylobacterium* isolates. The phylogenetic tree was constructed using approximately 500 bp nucleotide sequences of the *atpD* gene from 7 *Methylobacterium* isolates and the type strains belonging to the genus *Methylobacterium*. Bootstrap values are expressed in percentage based on 1,000 replications. *Methylobacterium* isolates HP2.1 (from Hau Giang); CP2.1, CP2.2, CP3.1, CP3.2 (from Can Tho); and VL2.1, VL2.2 (from Vinh Long).

(Lai *et al*., 2021)

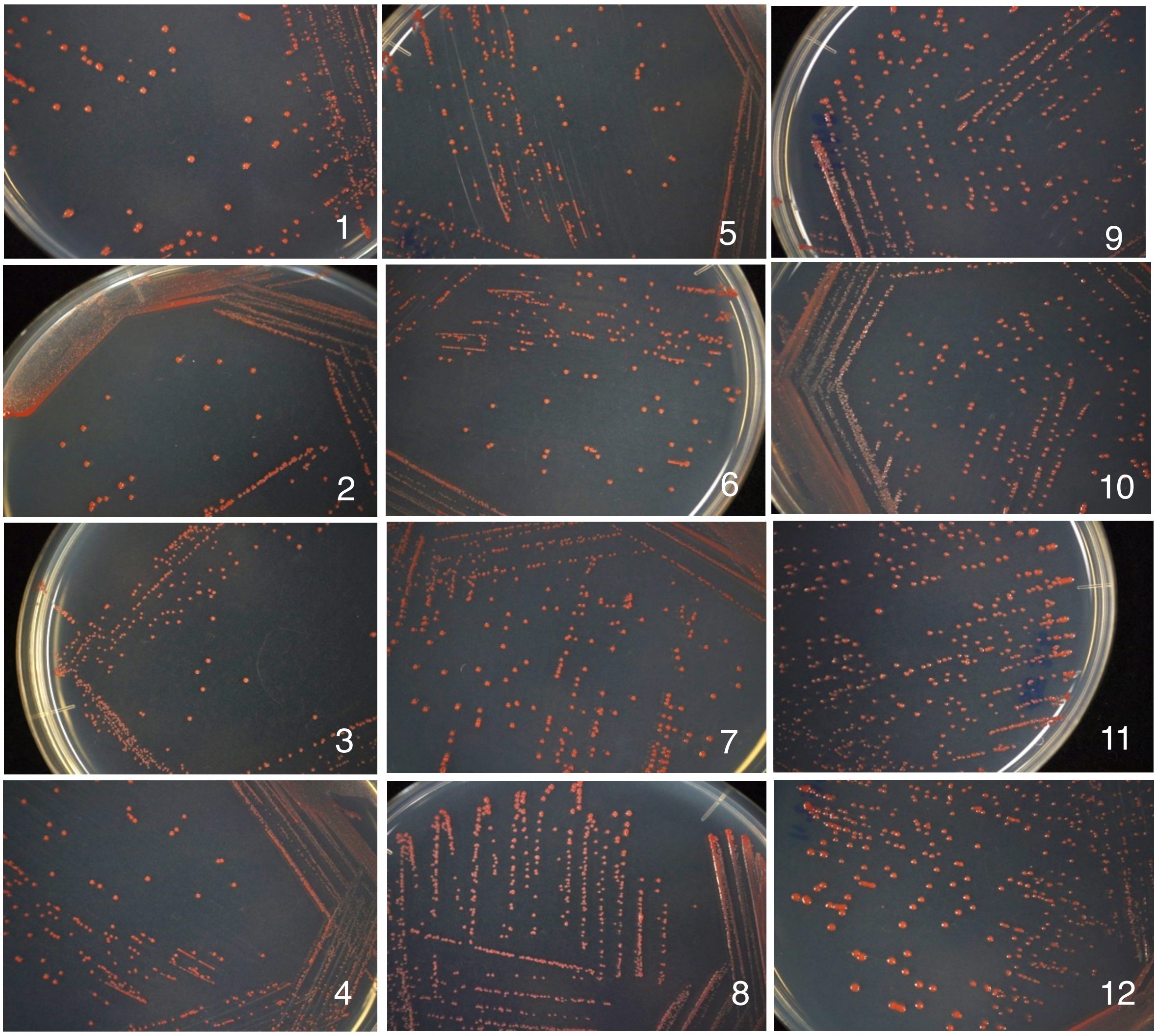

Figure S3. Colony morphology of the pathogenic *Methylobacterium* strains (1-10) and nonpathogenic *Methylobacterium* strains (11 and 12). 1. HP2.1, 2. CP2.1, 3. CP2.2, 4. CP3.1, 5. CP3.2, 6. VL2.1, 7. VL2.2, 8. VL1, 9. DP28.3, 10. DP28.4, 11. SE2.11^{T}, 12. SE3.6.

(Lai *et al*., 2021)

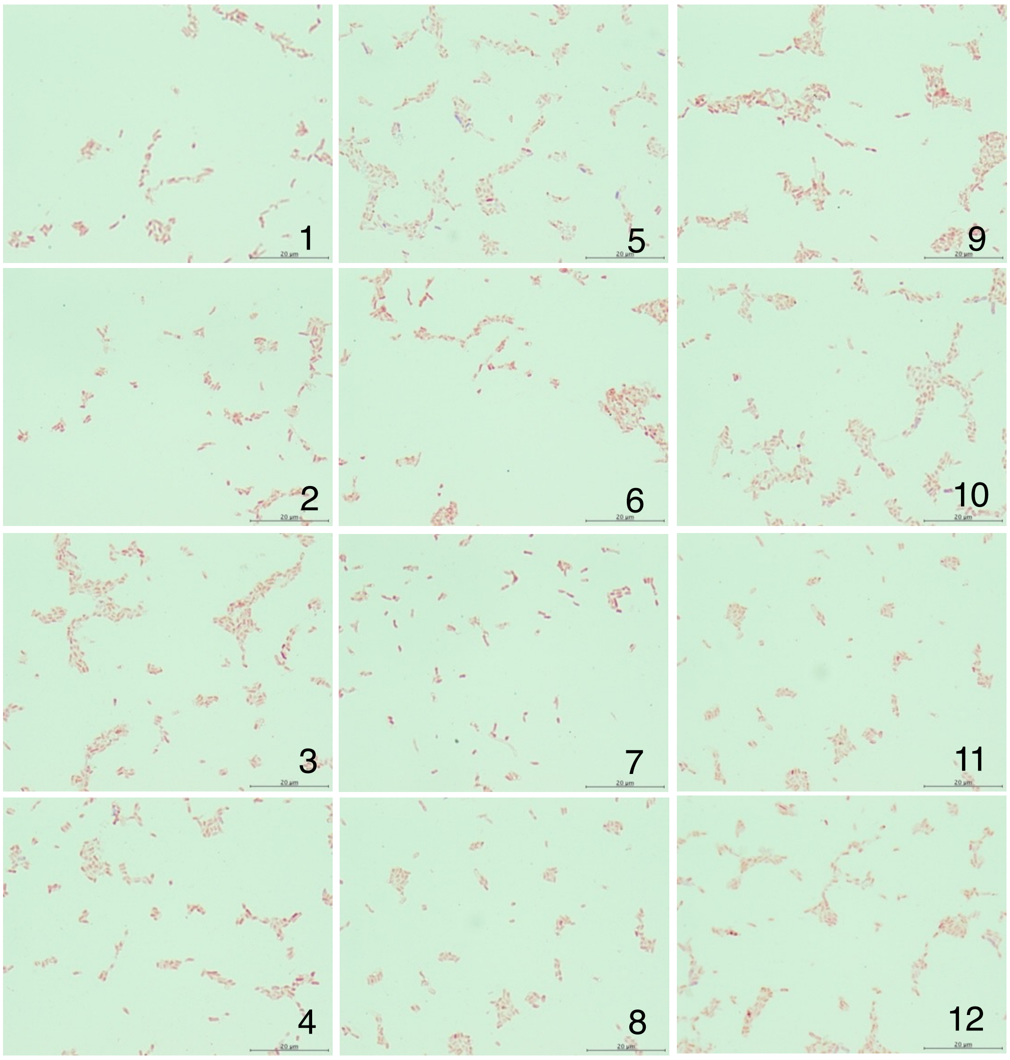

Figure S4. Microscopic examination of the pathogenic *Methylobacterium* strains (1-10) and nonpathogenic *Methylobacterium* strains (11 and 12) with Gram stain. 1. HP2.1, 2. CP2.1, 3. CP2.2, 4. CP3.1, 5. CP3.2, 6. VL2.1, 7. VL2.2, 8. VL1, 9. DP28.3, 10. DP28.4, 11. SE2.11^{T}, 12. SE3.6.

(Lai *et al.*, 2021)

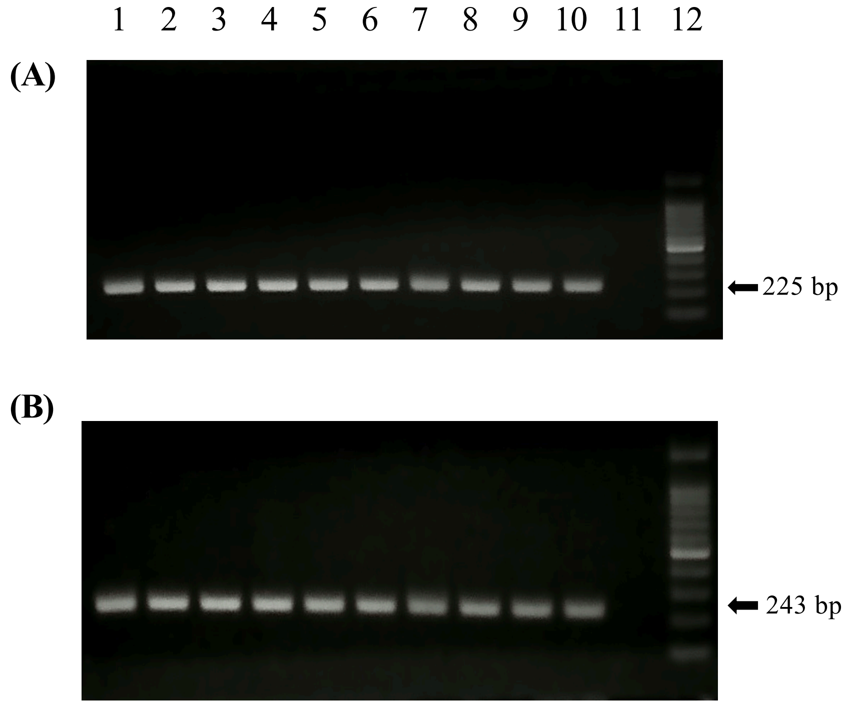

Figure S5. Detection the unique sequence 4 (A) and unique sequence 6 (B) in pathogenic *Methylobacterium* strains (lane 1-10) and nonpathogenic *Methylobacterium* strains (lane 11) by polymerase chain reactions (PCRs). 1. HP2.1, 2. CP2.1, 3. CP2.2, 4. CP3.1, 5. CP3.2, 6. VL2.1, 7. VL2.2, 8. VL1, 9. DP28.3, 10. DP28.4, 11. SE2.11^{T}. Lane 12, 100 bp DNA maker. PCR products were separated by a electrophoresis using a 1% TAE agarose gel and stained with ethidium bromide, then visualized using UV light. The arrow shows the position of the predicted PCR products.

(Lai *et al*., 2021)
